# GNN-FTuckER: A novel link prediction model for identifying suitable populations for tea varieties

**DOI:** 10.1371/journal.pone.0323315

**Published:** 2025-05-27

**Authors:** Jun Li, Bing Yang, Jiaxin Liu, Xu Wang, Zhongyuan Wu, Qiang Huang, Peng He

**Affiliations:** 1 College of Information Engineering, Sichuan Agricultural University, 46 Xinkang Road, Yucheng District, Ya’an, Sichuan province, China; 2 Agricultural Information Engineering Higher Institution Key Laboratory of Sichuan Province, Ya’an, Sichuan province, China; 3 Ya’an Digital Agricultural Engineering Technology Research Center, Ya’an, Sichuan province, China; 4 Institute of Agricultural Information and Rural Economy, Sichuan Academy of Agricultural Sciences, China; Air University, PAKISTAN

## Abstract

Current research on tea primarily focuses on foundational studies of phenotypic characteristics, with insufficient exploration of the relationship between tea varieties and suitable populations. To address this issue, this paper proposes a link prediction model based on Graph Neural Networks (GNN) and tensor decomposition, named GNN-FTuckER, designed to predict the “tea suitability” relationships within the tea knowledge graph. This model integrates the SE-GNN structural encoder with an improved TuckER model decoder. The SE-GNN encoder enhances the modeling capability of the global graph structure by explicitly modeling relations, entities, and triples, thereby obtaining embedding vectors through aggregation, updating, and iterative operations. The improved TuckER model enhances the capture of complex semantics between entities and relations by introducing nonlinear activation functions. To support our research, we constructed a tea dataset, TeaPle. In comparative experiments, GNN-FTuckER achieved superior performance on both public datasets (WN18RR, FB15k-237) and the TeaPle dataset. Ablation studies indicate that the model improved H@10 by 4.3% on the WN18RR dataset and by 1.5% on the FB15k-237 dataset, with a 1.3% increase in MRR. In the TeaPle dataset, H@3 improved by 4.7% and H@10 increased by 3.1%. This research provides significant insights for further exploring the potential of tea varieties and evaluating the health benefits of tea consumption.

## Introduction

Tea (Camellia sinensis, Theaceae) was first consumed over 1,500 years ago in China, primarily for medicinal purposes, particularly in Yunnan Province [1]. Today, tea has become the second most consumed beverage worldwide, after water, with its consumption expected to rise due to its aroma and flavor [2–4]. Numerous studies have confirmed the health benefits of tea [5]. For instance, Tang et al.[6] reviewed the literature on tea’s bioactive compounds, bioavailability, and health functions, concluding that tea provides numerous health advantages. Similarly, Sae-Tan et al. [7] demonstrated through in vitro and animal studies the potential of tea in preventing metabolic syndrome (MetS). However, improper tea consumption can pose health risks. Schönthal et al. [8] reported liver disease cases associated with green tea extract consumption, while Pillukat et al. [9] documented acute hepatitis caused by concentrated green tea extract. Thus, researching the characteristics of different tea types and their suitability for various populations is crucial.

Globally, over 3,000 types of tea exist. In China, tea is categorized into six major types: green tea, black tea, oolong tea, dark tea, yellow tea, and white tea. Key indicators like attributes, suitability, and health benefits are vital for determining which teas are appropriate for different populations [10]. In a randomized controlled trial, Mahdavi et al. [11] found that green tea has a stronger antihypertensive effect than black tea. Yan et al. [12] analyzed the composition and value of winter tea, promoting further research into its properties. Lee et al. [13] developed a tea recommendation system, TeaPick, validated through consumer acceptance tests. However, these studies largely rely on traditional statistical analyses, limiting the comparability of the data.

Recently, advancements in deep learning have prompted researchers to apply these techniques to tea studies. Chen et al. [14] used Convolutional Neural Networks (CNNs) and Gated Recurrent Units (GRUs), combined with the NCA algorithm, to predict moisture content and quality in Pu’er tea based on image data and EP. Xu et al. [15] proposed a two-stage fusion network for detecting and classifying tea buds, achieving an accuracy of 95.71%. Nevertheless, these studies focus primarily on external visual characteristics of tea plants, lacking comprehensive analysis of the six tea types and their associations with different populations.

In 2012, Google introduced the concept of the Knowledge Graph (KG) [16], which effectively integrates fragmented information across fields. KGs have been successfully applied in agricultural knowledge services and pest diagnosis [17]. For instance, Chen et al. [18] developed AgriKG, an agricultural knowledge graph for entity search and question answering. Given the vast data on tea, KGs provide a flexible structure to organize and store information, representing the complex relationships between tea varieties and suitable populations. This study utilizes KG technology to explore these relationships.

Link prediction is a core task in KG completion, inferring new relationships from existing ones to build more comprehensive graphs. Rossi et al. [19]. categorized link prediction models into tensor decomposition, translation, and neural network-based approaches. The TuckER model [20] uses tensor operations to capture complex interactions between entities and relationships, while the RotatE model [21] maps relationships as rotations in a complex vector space, effectively modeling intricate relationships. Although tensor and translation models offer flexibility and interpretability, they fall short in capturing deep semantic features, particularly in the complex network of relationships between tea varieties and target populations. Neural network-based models, in contrast, generate expressive embeddings, making them more suited to such complex data structures [22]. For instance, Dettmer et al. [23] introduced ConvE, a multilayer convolutional network for link prediction that achieved state-of-the-art performance. Vashishth et al. extended this with InteractE, improving feature interaction though compromising embedding structure. Graph Neural Networks (GNNs) like R-GCN [24] and CompGCN [25] capture rich semantic information between entities by considering neighborhood information and multi-relational data, outperforming traditional models. However, while these neural network-based models improve performance, they lack the flexibility and interpretability of tensor models.

Link prediction has been widely applied in various fields. For example, McCoy et al. [26] developed an end-to-end machine learning pipeline to predict missing links in biomedical literature for drug discovery, while Huo et al. [27] proposed a personalized social influence link prediction method for predicting user links in social networks. Nasiri et al. [28] introduced a feature selection-based random walk method to improve link prediction in protein-protein interactions. Despite these advances, link prediction research in tea studies remains underexplored. Existing research, such as that by Pan, Mahdavi, Yan, Lee, and Chen, focuses primarily on quality testing or internal composition, with limited exploration of the relationship between tea varieties and suitable populations.

In light of the above, while tensor models offer strong flexibility and interpretability, they lack the ability to capture contextual information. GNNs, by aggregating neighborhood information, excel in handling complex multi-relational data structures but are less flexible and interpretable than tensor models. Inspired by SE-GNN [29], this study proposes GNN-FTuckER, which integrates SE-GNN as an encoder into the original TuckER model [20] to explicitly model relationships, entities, and triples in KGs, capturing rich semantic information. We further enhance the TuckER decoder with a nonlinear activation function to better model the nonlinear relationships between entities and relations, generating more expressive feature representations. The proposed model combines the context-aware capabilities of GNNs with the interpretability of TuckER. Our experimental results demonstrate that GNN-FTuckER effectively addresses two key limitations of TuckER: its inability to leverage graph structure information and its reliance on tensor decomposition, which restricts its ability to model complex relationships.

In summary, the main contributions of this paper are:

- Propose the GNN-FTuckER model, combining the SE-GNN encoder and an improved TuckER decoder to address TuckER’s inability to capture contextual information in KGs..
- Introduce a nonlinear activation function to enhance TuckER’s capacity for modeling nonlinear relationships between entities and relations.
- Constructed a dataset with 6,698 records, including 330 tea varieties and 29 relations, to predict the “suitable population” relation and complete the tea KG.

## Method

### General

**Fig 1:**
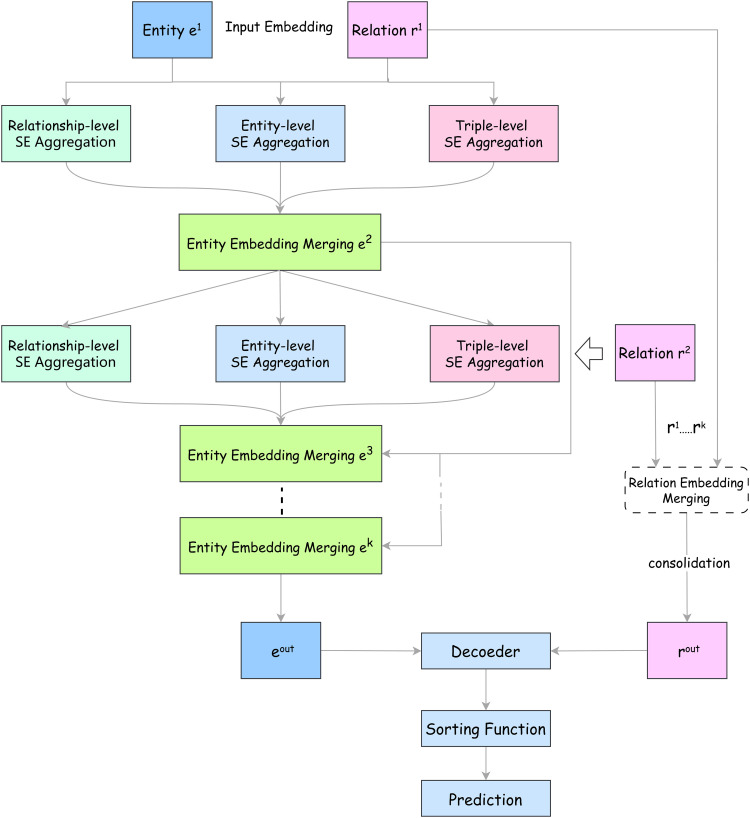
Structure of the GNN-FTuckER model. Note: The model consists of an input layer, aggregation layer, entity embedding fusion layer, relation embedding fusion layer, and output layer.

The architecture of the GNN-FTuckER model, shown in Fig 1, consists of two main components: the GNN encoder and the FTuckER decoder. This model addresses the original TuckER model’s limitations in capturing contextual information and its inability to learn deeper semantic features, while retaining its flexibility and interpretability.

First, the SE-GNN encoder explicitly models the knowledge graph at three semantic levels—relations, entities, and triples—by employing operations such as aggregation, updating, and recursive stacking. This process generates embedding vectors enriched with semantic information and graph structure. It effectively compensates for the TuckER model’s shortcomings in fully leveraging graph structure information. While the GNN model utilizes information propagation and aggregation across graph nodes to capture connections between entities, the TuckER model primarily focuses on tensor decomposition of entities and relations, limiting its ability to model complex entity relationships.

To address this, we introduced nonlinear activation functions to improve the TuckER model, allowing it to capture the inherently nonlinear interactions between entities and relations. This enhancement enables the model to learn more complex and expressive feature representations. Finally, the improved TuckER model is used for decoding and applied in link prediction experiments.

### GNN encoding structure

The core concept of Graph Neural Networks (GNNs) lies in learning node representations by capturing the relationships between nodes, enabling meaningful inferences and predictions over graph structures. GNNs operate through three key steps: Aggregation: GNNs aggregate information from neighboring nodes to incorporate broader, more global information. Update: Aggregated information is used to update the node representations by merging features from the node itself with those from its neighbors. Iteration: The aggregation and update steps are performed iteratively. Through multiple iterations, each node progressively gathers information and refines its representation.

**Fig 2:**
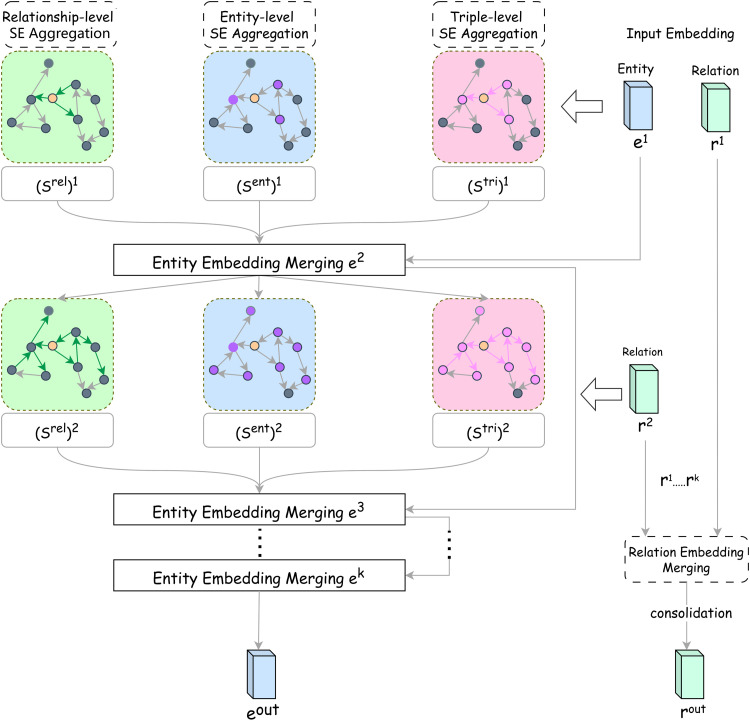
The architecture of the encoder. Note: Green, blue, and pink denote the three stages of SE aggregation for relations, entities, and triplets, respectively. The orange node represents the center node for neighbor aggregation.

In this paper, we employ the GNN layers from SE-GNN (Semantic Evidence-aware Graph Neural Network) as the encoder, as depicted in Fig 2. SE-GNN captures semantic evidence across three levels—relation, entity, and triplet—by explicitly modeling these as embedding vectors, allowing it to infer richer semantic relationships.

In knowledge graph inference, effective models typically learn rich semantic correlations during training. For link prediction, queries usually consist of known entities and relations, and the model predicts missing entities or infers new relations. SE-GNN summarizes query semantics into three categories:

#### Relationship level.

If the tail entity $\;\;\;\;{\text{e}}_{\text{o}}\;\;\;\;$ frequently appears in queries related to relation r, it can be inferred that r contains sufficient information to predict e $\;\;\;\;{\text{e}}_{\text{o}}$. For example, for the query “(Mao Lu, Production Location) →?”, regardless of variations in the head entity “Mao Lu (a tea name),” the probability of the tail entity being “China” is higher than it being “male.” The semantic interaction for the relation level is expressed by aggregating all connected relations, as shown in Equation (1):


$$s_{i}^{rel}=\sigma \left(\sum \left(e_{j},r_{j}\right)\in {𝒩}_{i}\alpha_{ij}^{rel}W^{rel}r_{j}\;\right).$$ (1)

Here, ${𝒩}_{\text{i}}$ represents the set of neighbors connected to ${\text{e}}_{\text{i}}$, ${\text{W}}^{\text{rel}}$ is the linear transformation matrix, and αijrel is the attention weight for relation aggregation:


$$\alpha_{ij}^{rel}=\frac{\exp\left(r_{j}^{T}e_{i}\right)}{\sum \left(e_{k},r_{k}\right)\in {𝒩}_{i}\;\;\exp\left(r_{k}^{T}e_{i}\right)}.$$ (2)

Where $e_{\text{i}}\in \;\;\;\;{\text{R}}^{\text{n}}\;\;\;\;$ represents the embedding of entity $e_{\text{i}}$. We utilize the dot product to dynamically compute the attention weight of neighboring relation $r_{\text{j}}$ concerning the central entity $e_{\text{i}}$, thereby assessing its importance.

#### Entity level.

If there is an indirect query path from $e_{\text{s}}$ to $\;\;\;\;e_{\text{o}}$ in the training set, it aids the direct inference between $e_{\text{s}}$ and $e_{\text{o}}$. For example, the known queries “( $e_{\text{s}}$, Produces) $\to e_{\text{i}}$ ” and “( $e_{\text{i}}$, Contains) $\to e_{\text{o}}$ ” can assist in predicting the query “( $e_{\text{s}}$, Produces) $\to e_{\text{o}}$.” The semantic interaction for the entity level is expressed by aggregating all connected neighbors, with the single-layer entity aggregation defined as:


$$s_{i}^{\text{ent}}=\sigma \left(\sum \left(e_{j},r_{j}\right)\in {𝒩}_{i}\;\alpha_{ij}^{\text{ent}}W^{\text{ent}}e_{j}\right)$$ (3)

Here, αijent  is the attention weight for entity aggregation, defined as:


$$\alpha_{ij}^{\text{ent}}=\frac{exp\left(e_{j}^{T}e_{i}\right)}{\sum \left(e_{k},r_{k}\right)\in {𝒩}_{i}\;\;exp\left(e_{k}^{T}e_{i}\right)}$$ (4)

#### Triple level.

If the query “( $e_{s}$, r) $\to e_{\text{o}}$ ” has been trained and a similar query exists, it aids the inference for query “ $\left(e_{s},r\right)$ $\to e_{o}^{\;\prime \;\;}$ ”. For example, the query “(Xiao Ming, Occupation) Tea Producer and Manufacturer” helps infer “(Xiao Ming, Occupation) Tea Seller.” SE-GNN captures these semantic correlations, referred to as semantic evidence (SE). Triplet-level aggregation considers both adjacent entities and relations, defined as:


$$s_{i}^{tri}=\sigma \left(\sum \left(e_{j},r_{j}\right)\in {𝒩}_{i}\alpha_{ij}^{tri}W^{tri}\varphi \left(e_{j},r_{j}\right)\;\right)$$ (5)

Here, $\;\;\varphi \;\;\left(e_{\text{j}},r_{\text{j}}\right)$ represents the combination function for aggregating entity and relation, and αijtri is the attention weight for triplet aggregation:


$$\alpha_{ij}^{tri}=\frac{exp\left(\varphi {\left(e_{j},r_{j}\right)}^{T}e_{i}\right)}{\sum \left(e_{k},r_{k}\right)\in {𝒩}_{i}\;\;exp\left(\varphi {\left(e_{k},r_{k}\right)}^{T}e_{i}\right)}$$ (6)

For multi-layer iterations, the aggregated embeddings are added to the original embeddings for the next layer’s input. By applying different relation-specific linear transformations across layers, the model yields the final output relation matrix.

### FTuckER decoder

**Fig 3:**
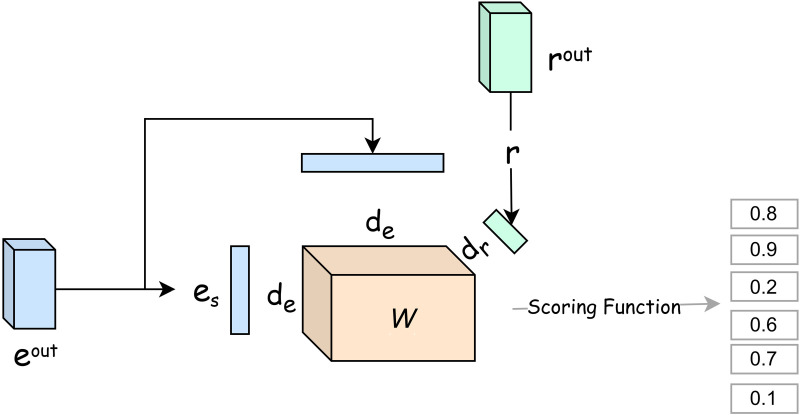
Decoder architecture. Note: The entity and relation embeddings produced by the encoder are fed into the FTuckER decoder for extrapolation. ${\text{e}}_{\text{s}}$ is the head entity vector, 𝒲 is the core tensor, r is the relation vector, and ${\text{e}}_{\text{o}}$ is the tail entity vector. Here, ${\text{e}}_{\text{s}},{\text{e}}_{\text{o}}\in {\text{R}}^{{\text{d}}_{\text{e}}},r\in {\text{R}}^{{\text{d}}_{\text{r}}}$, $r\in {\text{R}}^{{\text{d}}_{\text{r}}}$, and the core tensor 𝒲 $\in {\text{R}}^{{\text{d}}_{\text{e}}\cdot {\text{d}}_{\text{r}}\cdot {\text{d}}_{\text{e}}}$ is determined by the dimensions of both entities and relations.


In link prediction, the primary goal is to accurately score all missing triples. Scoring functions can be broadly categorized into linear structures and more complex neural network models with non-linear structures.As shown in Fig 3, the TuckER model uses third-order tensor decomposition to capture the interaction between entities and relations. Its scoring function is defined in Equation (7):

$$f_{r}\left({\text{e}}_{\text{s}},{\text{e}}_{\text{o}}\right)=\text{W}\cdot {\text{x}}_{1}\cdot e_{\text{s}}\cdot {\text{x}}_{2}\cdot r\cdot {\text{x}}_{3}\cdot e_{\text{o}}.$$ (7)

Where W is the shared parameter, ${\text{x}}_{1}$ represents the tensor product of the nth mode.

In knowledge graphs, the relationships between entities and relations are often non-linear. Non-linear models typically employ activation functions to introduce non-linear characteristics. To better capture these non-linear relationships and enhance model performance and generalization, this paper incorporates non-linear activation functions into the TuckER model. On one hand, this boosts the model’s representational power, allowing it to learn more complex and richer feature representations by capturing intricate semantic relationships. On the other hand, non-linear activation functions increase the model’s adaptability, making it more capable of handling diverse relationships and entities in knowledge graphs. Knowledge graphs often contain various entity types and relationships with highly non-linear connections, and adding non-linear activations helps improve both performance and generalization.

In summary, introducing non-linear activation functions allows the model to overcome the limitations of linear models and better represent complex relationships within knowledge graphs. We refer to this enhanced TuckER model with non-linear activations as FTuckER, with the updated scoring function shown in Equation (8):


$$f_{r}\left(e_{s},e_{o}\right)=f_{non}\left(\;\;\;\;\text{ W}\cdot {\text{x}}_{1}\cdot e_{s}\cdot {\text{x}}_{2}\cdot r\right){\text{x}}_{3}\cdot e_{o}.$$ (8)

Where $f_{non}$ *re*presents the non-linear activation function.

### Model scoring function

The overall structure of GNN-FTuckER is shown in Fig 1. By utilizing the GNN in SE-GNN, which aggregates three types of semantic information as the encoder, the model captures rich semantic embeddings. The incorporation of GNN allows the model to account for local structural information between entities, thereby better capturing global relationships within the knowledge graph. Combined with FTuckER, which has strong non-linear expressiveness as the decoder, the model’s capacity to handle complex semantic relationships between entities and relations is enhanced.

The scoring function for GNN-FTuckER is defined as follows Equation (9):


$$f_{r}\left(e_{s},e_{o}\right)=e_{o}^{T}\cdot f_{non}\left(\;\;\;\;\text{ W}\cdot {\text{x}}_{1}\cdot e_{gnn}\cdot {\text{x}}_{2}\cdot r_{gnn}\right).$$ (9)

Where $e_{gnn}$ and $r_{gnn}$ represent the entity and relation embedding vectors encoded by the GNN.

In summary, GNN-FTuckER is an end-to-end model that introduces both non-linear activation functions and GNNs into the TuckER framework. This allows it to better capture the non-linear, local, and global relationships between entities and relations in knowledge graph link prediction tasks, thereby improving prediction performance.

## Dataset and evaluation metrics

### Overview

This study utilizes three datasets. The TeaPle dataset, specifically curated for the research on “Tea Variety Suitability for Different Populations,” was meticulously collected and organized. The WN18RR and FB15k-23 datasets are public datasets, employed to evaluate the generalization performance of the GNN-FTuckER model. Details of the parameters “entities,” “relations,” and “triplets” for the three datasets are provided in Table 1:

**Table 1:** Overview of datasets used in the experiments.

Dataset	Entities	Relations	Triples
TeaPle	1064	28	6698
WN18RR [23]	40943	11	93003
FB15k-23 [30]	14541	237	310116

Note: The datasets differ significantly in scale and complexity, encompassing varying numbers of entities, relations, and triplets.

### Construction of TeaPle

**Fig 4:**
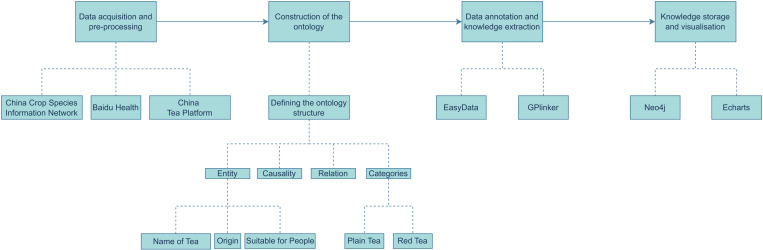
Top-down approach for constructing the tea knowledge graph. Note: The dataset construction process includes steps such as data collection and preprocessing, ontology development, data annotation and knowledge extraction, as well as storage and visualization.

The TeaPle dataset represents a vertical knowledge graph in the domain of tea, encompassing relevant terminologies, entities, relations, and attribute information. The construction process follows a top-down approach, where domain experts define the ontology to ensure its professionalism and accuracy. The overall workflow is shown in Fig 4 and proceeds as follows: Data Collection and Preprocessing: Tea-related data was collected using web scraping techniques from platforms such as the “China Crop Germplasm Information Network,” “China Tea Platform” (https://chayepingtai.com/?cate=3), and “Baidu Health.” After data cleaning, 6771 text-based documents were obtained. Ontology Construction: Entities, categories, attributes, and relations were defined. The tea-related entities include names, production regions, and target populations. Tea categories cover six main types: green tea, black tea, oolong tea, white tea, yellow tea, and dark tea. Data Annotation and Knowledge Extraction: Using the EasyData platform (https://console.bce.baidu.com/easydata), data was annotated, and entities and relations were extracted using a method based on GPlinker [31]. Manual disambiguation was conducted to improve accuracy, during the dataset annotation process, we implemented a structured labeling approach to ensure standardized data classification and annotation. To mitigate potential biases introduced by subjective judgment, we further employed cross-validation and other validation strategies, resulting in a final set of 6698 triples. Knowledge Storage and Visualization: The triples were stored using the Neo4j database [32], and the Echarts framework was used for visualization, enabling efficient querying and analysis of tea-related knowledge.

**Fig 5:**
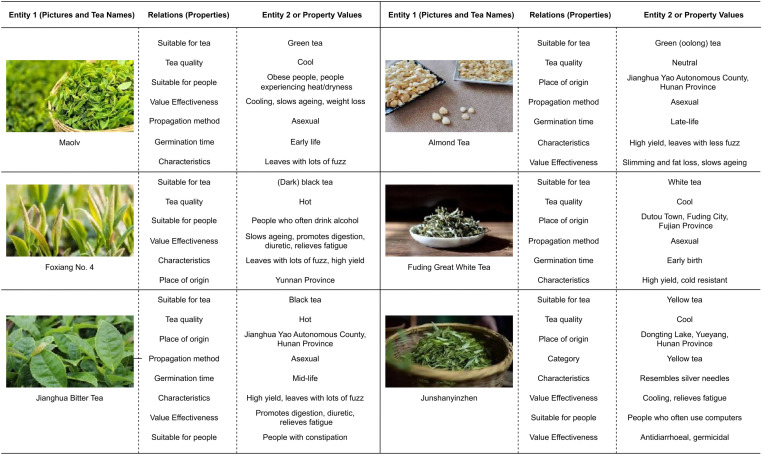
Example attributes or relations for six tea categories. Note: This presents information for six types of tea, including images, names, attributes, and suitable populations.

TeaPle also integrates characteristics and health benefits of various teas, categorized into 12 target populations commonly identified on the “Baidu Health” platform. These include individuals with obesity, smokers, those with damp-heat or dryness symptoms, people prone to cold, individuals with constipation or digestive issues, fatigue-prone individuals, alcohol consumers, people who frequently eat greasy foods, individuals with high blood pressure, high blood sugar, or high cholesterol, those with weakened immune systems, and frequent computer users. Examples are provided in Fig 5.

**Fig 6:**
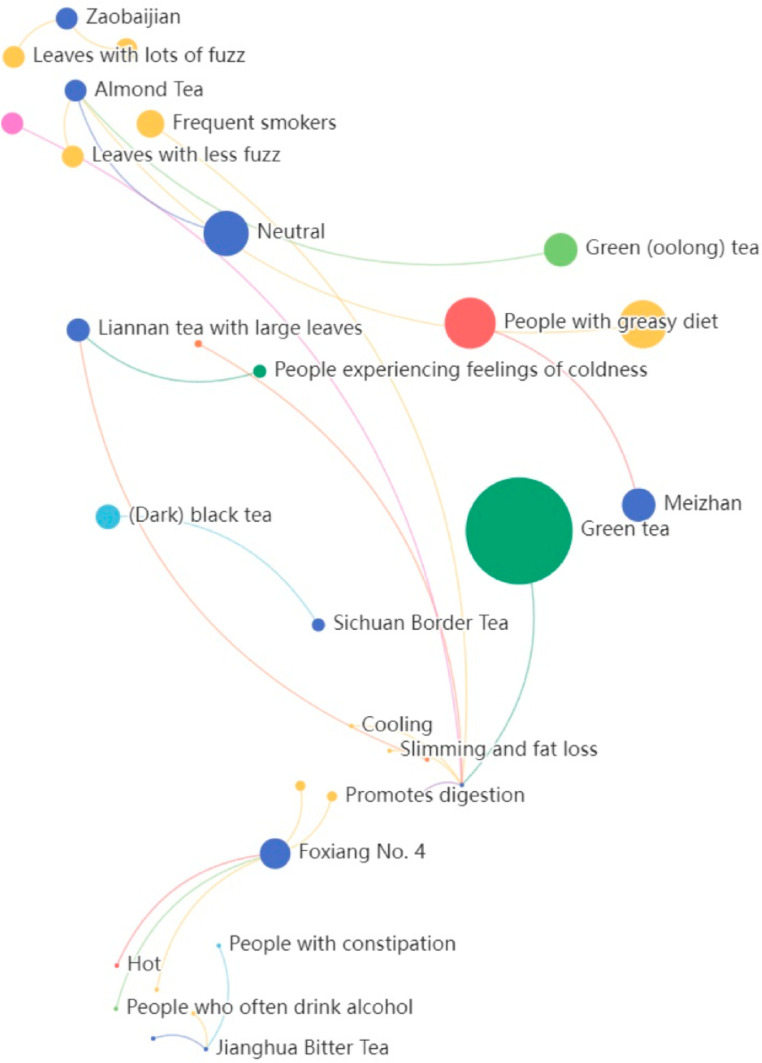
Visualization of the tea knowledge graph. Note: The graph displays information on different tea varieties, including their characteristics and suitable populations, through nodes and connections.

To provide a clear visual representation of the constructed knowledge graph, selected portions of the stored graph were visualized, as shown in Fig 6.

### Public dataset samples

WN18RR is a refined version of the WN18 dataset, derived from WordNet. It addresses the issue of test leakage in the original dataset by removing inverse relations. WN18RR consists of 40,943 entities, 11 relations, and approximately 93,003 triples. FB15k-237, sourced from Freebase, improves upon the FB15k dataset by removing redundant and inverse relations. It contains approximately 14,541 entities, 237 relations, and 310,116 triples.

### Evaluation metrics

In this study, we employ several standard metrics for link prediction tasks to evaluate the model’s performance, namely Hit@k, Mean Rank (MR) [33], and Mean Reciprocal Rank (MRR) [34].

Hit@k (H@k) measures whether the true link (positive example) is ranked among the top *k* predictions, where *k* is a specified integer, typically set to 1, 3, or 10. It evaluates the model’s ability to correctly rank the true triplet higher. The value of H@k ranges from 0 to 1, with higher values indicating better performance. The formula for H@k is as follows:


$$H@k=\frac{1}{\left|N\right|}\sum_{t=1}^{\left|N\right|}\;I\left({rank}_{t}⩽k\right)$$ (10)

Mean Rank (MR) is used to assess the model’s average rank for all predictions. For each query or test instance, MR calculates its corresponding rank and then averages the ranks across all instances. The range of MR is positive integers, where smaller values indicate better ranking performance. The formula for MR is given as:


$$MR=\frac{1}{\left|N\right|}\sum_{t=1}^{\left|N\right|}\;rank_{t}$$ (11)

Mean Reciprocal Rank (MRR) evaluates the quality of the model’s ranking by considering the reciprocal of the rank for each test instance. MRR ranges from 0 to 1, where values closer to 1 indicate better ranking performance. The formula for MRR is as follows:


$$MRR=\frac{1}{\left|N\right|}\sum_{t=1}^{\left|N\right|}\;\frac{1}{{rank}_{i}}$$ (12)

In these formulas, N represents the set of evaluated triplets, |N| is the total number of triplets, and $\text{ran}{\text{k}}_{\text{i}}$ refers to the rank of the ith triplet in the link prediction task. The indicator function 𝕀 evaluates to 1 if the condition $\text{ran}{\text{k}}_{\text{i}}\leq \text{k}$ holds true, and 0 otherwise.

## Experiments and evaluation metrics

### Overview

This study proposes a novel link prediction model based on Graph Neural Networks (GNN) and Tensor Factorization, called GNN-FTuckER. To comprehensively evaluate the performance of the proposed GNN-FTuckER model, a series of experiments were conducted on three datasets: TeaPle and two public datasets (WN18RR, FB15k-237). Specifically, the TeaPle dataset addresses the challenge of selecting appropriate populations for underrepresented tea varieties, while the public datasets are used to validate the effectiveness and generalization performance of the GNN-FTuckER model. The main experimental components include the following five parts:

- Comparison of GNN-FTuckER with classical link prediction models on TeaPle..
- Ablation study of GNN-FTuckER on TeaPle.
- Qualitative analysis of GNN-FTuckER on TeaPle.
- Comparison of GNN-FTuckER with classical link prediction models on public datasets (WN18RR, FB15k-237).
- Ablation study of GNN-FTuckER on public datasets.

### Dataset splits and experimental parameters

The split details of the three datasets used in the experiments are provided in Table 2.

**Table 2:** Dataset splits used in the experiments.

Dataset	Total	Train	Val	Test
Teaple	6698	5368	665	665
WN18RR	93003	86835	3034	3134
FB15k-237	310116	272115	17535	20466

The experimental setup and parameters used in this study are summarized in Table 3. The experiments were conducted on an NVIDIA RTX 4090 GPU with Windows 10 as the operating system. Python 3.8 was used for programming, with PyTorch 1.10.0 as the deep learning framework and CUDA version 12.2. Model parameters include Xavier initialization, the Adam optimizer (adaptive learning rate), and binary cross-entropy loss. The model architecture consists of GNN layers with {1, 2, 3} layers and hidden dimensions of {100, 200}. Training was performed for 500 epochs with an early stopping mechanism (training halts if validation performance does not improve for 50 consecutive epochs). Hyperparameters include learning rates of {0.00035, 0.0015, 0.003, 0.005}, batch sizes of {128, 256, 512, 1024}, and dropout rates of {0.1, 0.2, 0.3, 0.5}. All random seeds were fixed to 1234 for consistency. It is important to note that, except for the results of some comparison models on public datasets, which were directly referenced from the original literature, all experimental parameters remained consistent across the model training.

**Table 3:** Experimental platform and parameter settings.

Parameter	Value	Parameter	Value
GPU	RTX4090	Learning rate	{0.00035,0.0015,0.001,0.003,0.005}
Pytorch	1.10.0	Batch size	{128,256,512,1024}
Software	Python 3.8	Gnn-layer	{1,2,3}
OS	Windows	H_dim	{100,200}
Epoch	500	Input_dropout	{0.1,0.2,0.3,0.5}
Early Stop	50	Hidden_dropout1	{0.1,0.2,0.3,0.5}
Loss Function	Binary cross-entropy	Hidden_dropout2	{0.1,0.2,0.3,0.5}
Initialization	Xavier [35]	Optimizer	Adam [36]
Cuda	12.2	Random_Seed	1234

### Experiments on TeaPle dataset

#### Comparative experiments.

The GNN-FTuckER model is primarily a neural network model. This section presents a comparison of its link prediction results on the TeaPle dataset with several established link prediction models. These include ConvE [23], which utilizes 2D convolutions on reshaped embeddings to capture entity-relation interactions; CompGCN [25], a graph convolutional network that integrates entities and relations to enhance link prediction; TransE [37], models relations via vector addition in the same embedding space for entities and relations. DistMult [38], uses a bilinear scoring function, suitable for symmetric relations. ComplEx [39], extends bilinear models to the complex space to handle antisymmetric relations. RotatE [21], models relations through rotational transformations in the complex space. pRotatE [21], extends RotatE by incorporating learnable phase representations, enhancing the model’s capability to capture relational patterns. InteractE [40], an advanced embedding model that optimizes entity-relation interactions through multiple feature rearrangements; TuckER [20], a tensor decomposition model that employs a three-way tensor representation to encapsulate multi-relational interactions; and SE-GNN [29], which harnesses semantic information to enhance knowledge graph embedding tasks.

The comparative experimental results, presented in Table 4, demonstrate the superior link prediction performance of the GNN-FTuckER model on the TeaPle dataset. It achieves an MRR that surpasses the best-performing baseline, SE-GNN, by 1.8%, along with a 1.0 improvement in the MR metric. The strong performance of GNN-FTuckER can be attributed to two key factors. First, GNN-FTuckER effectively models knowledge graphs across three semantic levels—relations, entities, and triples—by leveraging a GNN-based encoder. Through aggregation, updating, and stacking mechanisms, the model generates embeddings enriched with semantic information and structural awareness. This addresses a critical limitation of existing models, which often fail to directly utilize graph structural information, thereby missing important relational cues. Second, most existing models assume a linear association between entities and relations, employing linear activation functions. This oversimplifies the complex, nonlinear dependencies within knowledge graphs. In contrast, GNN-FTuckER integrates nonlinear activation functions, enhancing its ability to capture intricate relationships between entities and relations, further improving prediction accuracy.

**Table 4:** Comparison of GNN-FTuckER with classic link prediction models.

Model	MRR(%)	MR	H@1(%)	H@10(%)
RotatE	49.9	84.0	41.1	67.1
pRotatE	51.0	83.2	42.3	67.1
ComplEx	52.3	53.5	44.0	67.6
DistMult	58.3	52.8	50.3	73.7
TransE	58.9	48.9	52.7	70.1
ConvE	56.4	59.1	47.6	72.9
CompGCN	60.2	38.3	53.4	72.7
InteractE	59.6	42.3	52.9	72.5
TuckER	57.6	56.7	49.2	73.4
SE-GNN	62.8	21.6	56.9	75.2
GNN-FTuckER	**64.6**	**20.6**	**58.5**	**76.3**

Note: Bold and underlined values indicate the best and second-best performances, respectively. All experiments were conducted using the following hyperparameter settings: learning rate = 0.001, batch size = 256, number of layers = 1, embedding dimension = 200, input dropout = 0.1, and hidden dropout rates Hdrop1 = 0.2 and Hdrop2 = 0.3.

#### Ablation study.

In this set of experiments, the baseline model selected is the original TuckER model. Given that the TeaPle dataset may exhibit specific data characteristics and noise conditions not present in public datasets, performing an ablation study on the tea knowledge graph allows us to explore the robustness of GNN-FTuckER in the domain of tea knowledge graphs. This evaluation also assesses the model’s adaptability to noise and data variations, while confirming the effectiveness of the proposed improvements. The results of the ablation experiments are summarized in Table 5.

**Table 5:** Ablation performance of GNN-FTuckER on TeaPle dataset.

Model	GNN	$f_{non}$	MRR(%)	MR	H@1(%)	H@10(%)
TuckER	**×**	**×**	57.6	49.2	62.1	73.4
	**×**	**√**	58.3	50.2	62.7	73.4
	**√**	**×**	59.1	53.0	62.2	74.6
	**√**	**√**	**64.6**	**58.5**	**66.8**	**76.5**

Note: × indicates not introduced, while √ indicates introduced. All experiments in this group were conducted under the following hyperparameter settings: learning rate = 0.001, batch size = 256, layers = 1, embedding dimension = 200, input dropout = 0.1, and hidden dropout rates (Hdrop1 = 0.2, Hdrop2 = 0.3).

As shown in Table 5, the introduction of GNN layers and nonlinear activation functions significantly enhanced the performance of the baseline TuckER model. The best performance was achieved when both were incorporated. Notably, the TuckER model uses a linear activation function, which overlooks the inherent nonlinear interactions between entities and relations, especially in tasks like selecting suitable groups based on tea varieties. To address this issue, we introduced nonlinear activation functions to capture these inherent nonlinear interactions. The experimental results demonstrate that incorporating nonlinear activation functions effectively improved the model’s ability to learn more complex and expressive feature representations, resulting in a 0.7% improvement in the MRR metric. Furthermore, the TuckER model primarily focuses on tensor decomposition of entities and relations, limiting its ability to model complex entity relationships. In contrast, the GNN model propagates and aggregates information across graph nodes to capture connections between entities, directly addressing TuckER’s limitation in utilizing graph structure information. The inclusion of GNN resulted in a 1.5% improvement in the MRR metric.

#### Qualitative study.

In the qualitative analysis experiment, we first explored the potential impact of different embedding dimensions on the performance of GNN-FTuckER. Specifically, the choice of embedding dimension is a critical factor in model design, as it requires balancing information retention with computational and storage efficiency. Lower dimensions may lead to information loss, while higher dimensions increase computational and memory costs.

**Fig 7:**
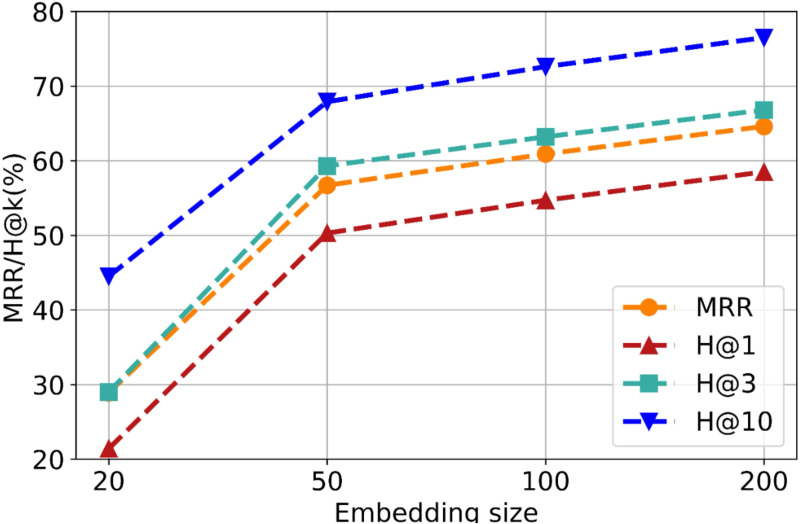
Performance of GNN-FTuckER with different embedding dimensions on the TeaPle dataset.

The results of the embedding dimension experiment are shown in Fig 7. It is evident that the most significant performance improvement occurs when the embedding dimension increases from 20 to 50, while the improvement becomes more gradual as it moves from 50 to 200. GNN-FTuckER achieved its best performance with an embedding dimension of 200, where the average probability of the target entity appearing in the top 10 predicted entities was 76.5%. Although larger embedding dimensions could potentially yield slightly better results, they also increase computational costs and pose challenges in training and deployment. Considering the trade-off between cost and performance, we set the embedding dimension of GNN-FTuckER to 200.

**Fig 8:**
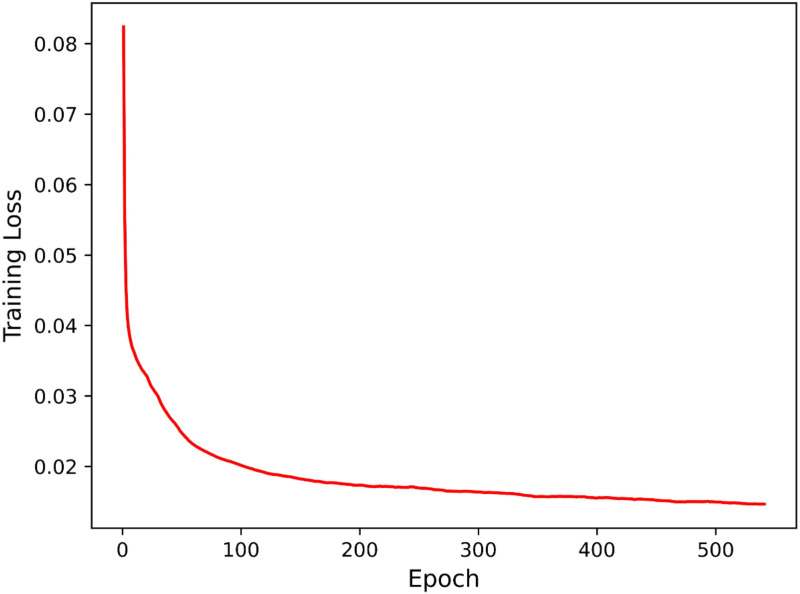
Training loss variation of GNN-FTuckER on the TeaPle dataset. Note: The training loss of the GNN-FTuckER model on the TeaPle dataset decreases gradually with the number of training epochs, indicating model convergence.

Furthermore, to further evaluate the performance of the GNN-FTuckER model on TeaPle, we plotted its training loss, as shown in Fig 8. The results indicate that around 300 epochs, the model exhibits stable convergence, with no signs of overfitting or underfitting.

Next, we compute the prediction scores using Equation 9. In knowledge graph completion tasks, it is common to score new triples and set an inclusion threshold. The selection of the inclusion threshold typically depends on the dataset’s characteristics, model performance, and domain expertise, and requires multiple iterations and domain knowledge for determination. For the tea knowledge graph, we ultimately determined that a high threshold of 0.9 is appropriate. Any triple with a score of 0.9 or higher is considered a “suitable overall” relation in the knowledge graph. The reason for choosing 0.9 as the threshold is that the tea knowledge graph involves complex domain knowledge, and only triples with very high scores can ensure accuracy and reliability, preventing erroneous triples from being incorporated. Additionally, a high threshold significantly reduces the false positive rate, minimizing the introduction of noisy data and thus enhancing the overall quality of the knowledge graph. Furthermore, through repeated experiments, we found that a threshold of 0.9 strikes a good balance between precision and recall, avoiding the introduction of too many low-confidence triples while ensuring sufficient coverage of the knowledge graph. Finally, as the tea knowledge graph needs to support high-precision queries and reasoning in practical applications, the 0.9 threshold meets this requirement, ensuring high-quality output.

**Fig 9:**
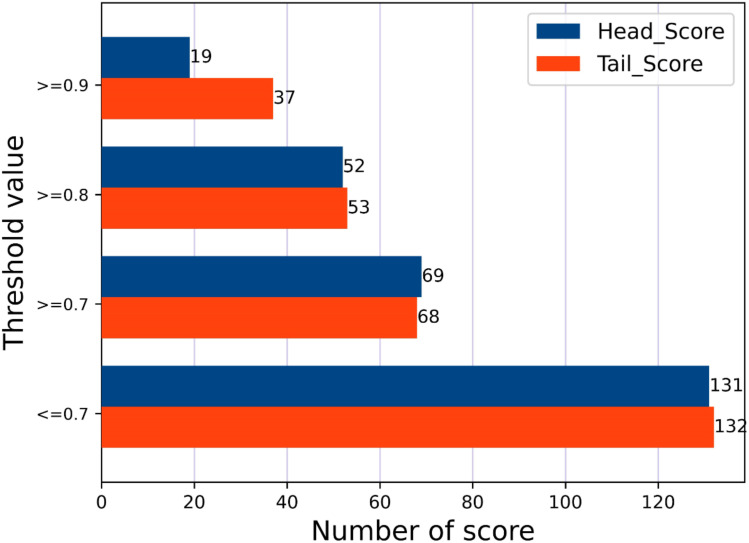
Score distribution of 200 predicted triples from GNN-FTuckER. Note: With different thresholds, the blue and orange bars represent the number of scores for the Head and Tail parts, respectively.

Additionally, we extracted all tea entities and known suitable population entities from the existing tea knowledge graph. By randomly combining each tea type with each suitable population, we generated new target triples, ensuring that these triples did not overlap with those already present in the knowledge graph. The purpose of this is to evaluate the model’s generalization ability and prediction accuracy in knowledge graph completion tasks, testing whether it can accurately predict unseen relations rather than just memorizing existing triples from the training data. We evaluated 200 predicted triples, with their score distribution shown in Fig 9. Table 6 provides some examples of the predicted triples and their scores.

**Table 6:** Sample predicted triples and their scores.

Prediction Triples	Scores
(Zaobaijian, suitable for people, obese people)	0.968
(Foxiang No. 4, suitable for people, people who suffer from three highs)	0.970
(Jianghua Bitter Tea, suitable for people, people experiencing feelings of coldness)	0.963
(Almond Tea, suitable for people, people with greasy diet)	0.936
(Sichuan Biancha (tea), suitable for people, people experiencing feelings of heat and dryness)	0.709
……	……

Note: Higher prediction scores indicate better matching performance of the model in identifying suitable populations for each tea type.

As shown in Fig 9 and Table 6, the majority of predicted triples have scores exceeding 0.9, further validating the model’s prediction consistency and the appropriateness of the threshold setting. This result indicates that the model possesses strong generalization ability, learning the underlying patterns between tea types and suitable populations from existing data, and making high-confidence predictions for new triples. Additionally, the high-scoring predictions provide a reliable foundation for expanding the knowledge graph, supporting the enrichment of its content and the discovery of potential new relations.

**Fig 10:**
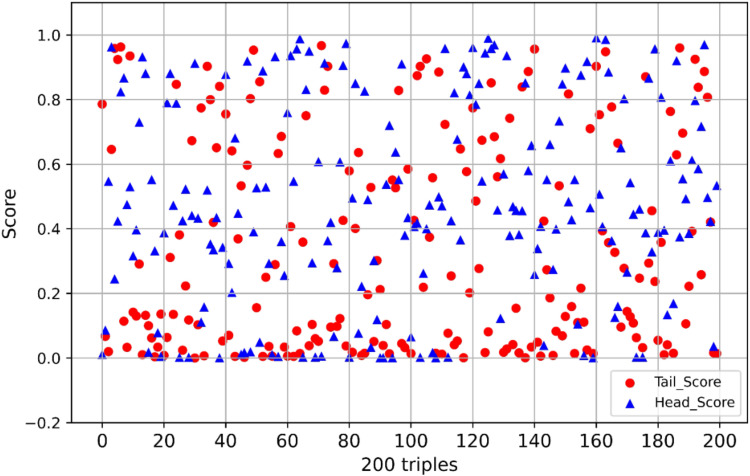
Scores of the 200 target triples. Note: The red circular markers represent Tail_Score, while the blue triangular markers represent Head_Score.

In Fig 10, “Head-batch” and “Tail-batch” refer to the scores of factual triples when predicting the head entity based on the relation and tail entity, respectively. Given that there are more tea types than suitable populations in the TeaPle dataset, we used the Head_Score as the final score for completing the knowledge graph triples.

**Fig 11:**
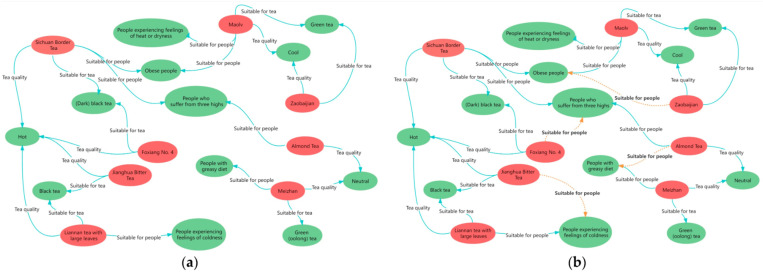
Updated knowledge graph after link prediction. Note: (a) represents the original knowledge graph, and (b) represents the completed knowledge graph. The green blocks denote the suitable populations and tea attributes, while the red blocks represent tea varieties. The orange dashed line indicates the relationship between the link’s predicted post-update.

Finally, we present a comparison of the tea knowledge graph before and after link prediction, as shown in Fig 11. It is evident that the predicted knowledge graph (b) effectively fills in the missing knowledge from the original graph (a). This improvement is attributed to the GNN-FTuckER model’s ability to accurately determine the “suitable population” relationships in the tea knowledge graph through the scoring function. The completed knowledge graph highlights the impact of tea on human health, provides tea recommendations, and facilitates accurate matching of supply and demand in tea production services, thus supporting decision-making.

### Experiments on public datasets

#### Comparative experiments on public datasets.

The objective of this set of experiments is to demonstrate the robustness and generalization ability of the proposed GNN-FTuckER model by comparing its performance on the two public datasets, WN18RR and FB15k-237, with several recent classical models. In addition to the models introduced in Method section, including ConvE, CompGCN, InteractE, TuckER, TransE, DistMult, ComplEx, RotatE and SE-GNN, the comparison also involves several other models: R-GCN [24]: Aggregates neighbor information for multi-relational reasoning using graph convolutional networks. KBGAN [41]: Enhances embedding training by generating negative samples with GANs. ConvTransE [42]: Applies convolutional operations to TransE for capturing local features. SACN [42]: Combines GCN and CNN for improved reasoning through end-to-end training.

The comparison results, as shown in Table 7, reveal that GNN-FTuckER outperforms most models on the WN18RR dataset. Although it ranks just below SE-GNN and CompGCN in the MRR, MR, and H@10 metrics, it still achieves the second-best performance on these three metrics and surpasses all comparison models on the H@1 metric. On the FB15k-237 dataset, GNN-FTuckER outperforms all comparison models across all metrics. Notably, GNN-FTuckER and FTuckER (which replaces the linear activation function in TuckER with a non-linear one) consistently outperform TuckER across all metrics on both WN18RR and FB15k-237. These results demonstrate that the GNN-FTuckER model not only excels on the TeaPle dataset but also maintains excellent performance on datasets with significant stylistic differences, showcasing its robustness and generalization capabilities.

**Table 7:** Link prediction performance comparison between GNN-FTuckER and classic models on WN18RR and FB15k-237.

Model	WN18RR				FB15k-237			
	MRR	MR	H@10	H@1	MRR	MR	H@10	H@1
TransE^*^	0.226	3384	0.501	—	0.330	173	0.528	0.231
DistMult^*^	0.430	5110	0.490	0.390	0.308	173	0.485	0.219
ComplEx^*^	0.440	5216	0.510	0.410	0.323	165	0.513	0.229
RotatE^*^	0.476	3340	0.571	0.428	0.338	177	0.533	0.241
R-GCN^*^	—	—	—	—	0.248	–	0.417	0.151
KBGAN^*^	0.214	—	0.472	—	0.278	–	0.458	—
ConvTransE^*^	0.460	—	0.520	0.430	0.330	–	0.510	0.240
SACN^*^	0.470	—	0.540	0.430	0.350	–	0.540	0.260
ConvE	0.430	4187	0.520	0.400	0.325	244	0.501	0.237
CompGCN	0.469	**3307**	0.536	0.434	0.355	197	0.535	0.264
InteractE	0.463	5202	0.528	0.430	0.354	172	0.535	0.263
TuckER	0.464	6681	0.517	0.438	0.354	165	0.536	0.262
SE-GNN(d = 200)	**0.491**	3402	**0.569**	0.447	0.340	166	0.515	0.251
FTuckER	0.475	5461	0.533	0.445	0.358	185	0.542	0.267
GNN- FTuckER	0.485	3688	0.560	**0.451**	**0.367**	**165**	**0.551**	**0.274**

Note: Bold, underline, and ‘-’ represent the best, second-best, and missing values, respectively. ‘

* ’ indicates results cited from the corresponding papers. The optimal hyperparameters for GNN-FTuckER on WN18RR are: lr = 0.003, batch = 256, ly = 1, d = 200, Idrop = 0.1, Hdrop1 = 0.2, Hdrop2 = 0.3. For FB15k-237, the optimal hyperparameters are: lr = 0.00035, batch = 1024, ly = 2, d = 200, Idrop = 0.1, Hdrop1 = 0.2, Hdrop2 = 0.3.

**Fig 12:**
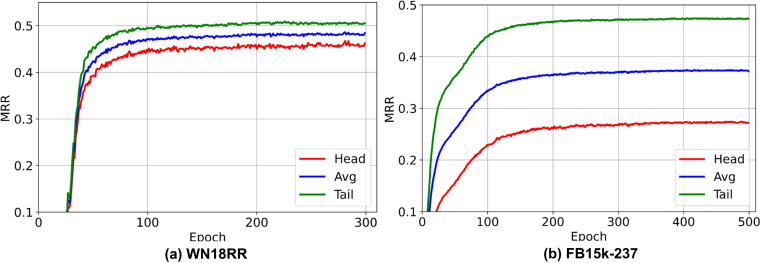
MRR convergence of GNN-FTuckER on WN18RR and FB15k-237. Note: (a) shows the MRR trend of GNN-FTuckER on the WN18RR dataset, and (b) shows the MRR trend of GNN-FTuckER on the FB15k-237 dataset.

Additionally, we plotted the performance of GNN-FTuckER in terms of MRR during training on WN18RR and FB15K-237, as shown in Fig 12. It can be observed that on the WN18RR dataset, the MRR value stabilizes around 100 epochs and triggers early stopping at 300 epochs. On the FB15k-237 dataset, the MRR stabilizes around 200 epochs. This demonstrates the strong generalization ability of GNN-FTuckER on public datasets, with no signs of overfitting or underfitting.

**Fig 13:**
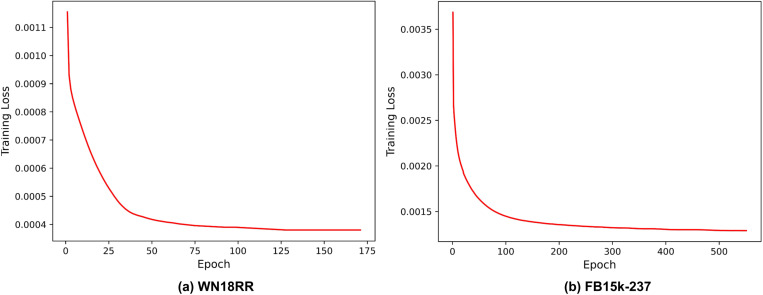
Training loss of GNN-FTuckER. Note: (a) Training loss trend of GNN-FTuckER on the WN18RR dataset; (b) Training loss trend of GNN-FTuckER on the FB15k-237 dataset.

Finally, to further observe the performance changes during training on public datasets, we plotted the training loss trends of GNN-FTuckER on both datasets, as shown in Fig 13. As seen, the training loss of GNN-FTuckER decreases rapidly from 0 to 100 epochs on both datasets, then stabilizes with a converging trend. For WN18RR, training is terminated early due to the truncation mechanism. Overall, the model exhibits no signs of overfitting or underfitting on either dataset and remains stable within a lower loss range.

In summary, compared to TuckER, GNN-FTuckER improves all evaluation metrics on the WN18RR dataset, with MRR increasing by 2.1% and H@10 by 4.3%. On the FB15k-237 dataset, GNN-FTuckER outperforms all comparison models across all metrics. Specifically, it improves H@10 by 1.5% compared to TuckER. These results demonstrate that GNN-FTuckER captures more global information and learns richer triple features, validating the effectiveness of the proposed improvements. Not only is the model effective on TeaPle, but it also shows significant performance gains on other datasets.

#### Ablation experiments.

In this section, we conduct ablation experiments on TuckER by introducing Graph Neural Networks (GNN) and a nonlinear activation function $f_{non}$. These experiments were conducted on two public datasets to demonstrate that the improvements brought by the GNN and nonlinear activation functions are not only effective on the TeaPle dataset, but also lead to significant performance gains on publicly available datasets. Tables 8 and 9 present a comparative analysis of TuckER’s metrics before and after the incorporation of the GNN layer and $f_{non}$ on the WN18RR and FB15k-237 datasets.

**Table 8:** Ablation experiments on the WN18RR dataset.

Model	GNN	$f_{non}$	MRR(%)	MR	H@10(%)	H@1(%)
TuckER	×	×	0.464	6681	0.517	0.438
	×	√	0.475	5461	0.533	0.445
	√	×	0.479	4435	0.547	0.446
	√	√	**0.485**	**3688**	**0.560**	**0.451**

Note: × indicates not introduced, while √ indicates introduced. $f_{non}$ refers to the linear activation function.

**Table 9:** Ablation experiments on the FB15k-237dataset.

Model	GNN	$f_{non}$	MRR(%)	MR	H@10(%)	H@1(%)
TuckER	×	×	0.354	165	0.536	0.262
	×	√	0.358	185	0.542	0.267
	√	×	0.348	167	0.532	0.257
	√	√	**0.367**	**165**	**0.551**	**0.274**

Note: × indicates not introduced, while √ indicates introduced. $f_{non}$ refers to the linear activation function.

The results in Tables 8 and 9 demonstrate that the GNN-FTuckER model, whether incorporating GNN or a nonlinear activation function, significantly improves link prediction performance on the public datasets (WN18RR and FB15k-237) compared to the original TuckER model. The best performance is achieved when both components are integrated. This further validates the effectiveness of the proposed improvements, not only on the TeaPle dataset but also on publicly available datasets with different characteristics.

**Fig 14:**
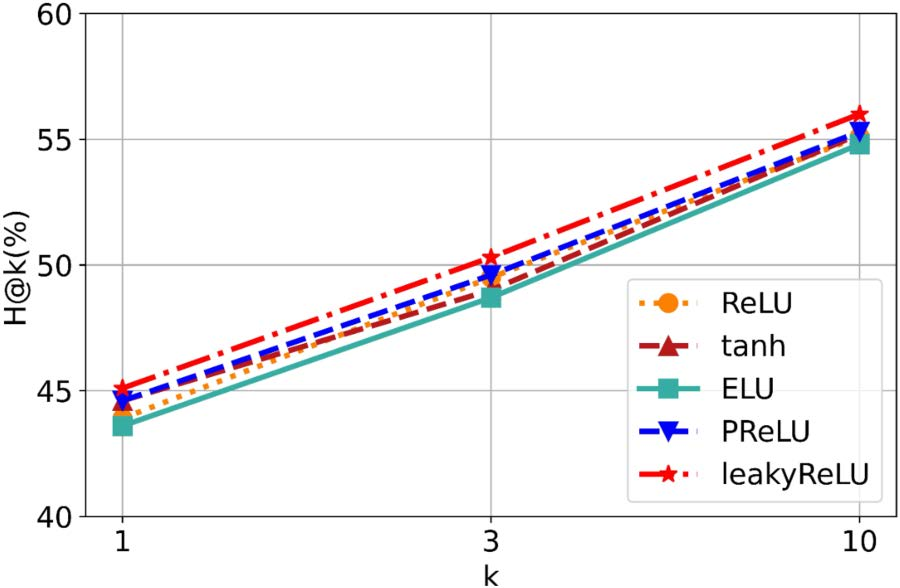
Comparison of H@n values for different activation functions in GNN-FTuckER on WN18RR.

Finally, to select the optimal nonlinear activation function, we replaced the activation function in the GNN-FTuckER framework with five common options —ReLU, tanh, ELU, PReLU, and leakyReLU—to compare the performance differences on WN18RR. As shown in Fig 14, leakyReLU consistently outperforms the others.

## Discussion

This study begins by analyzing the shortcomings in current tea research and introduces the development of knowledge graph technology in agriculture. Based on this foundation, we designed an end-to-end link prediction model—GNN-FTucER—to enhance the original TuckER model. The SE-GNN encoder effectively models knowledge graphs at three semantic levels: relations, entities, and triples, utilizing operations such as aggregation, updating, and stacking to obtain embedding vectors rich in semantic information and graph structure. This addresses the limitation of the original TuckER model, which inadequately captures information due to its lack of direct utilization of graph structural information. Consequently, we introduced the GNN layer as our encoder, enabling information propagation and aggregation on the graph while leveraging the connections between nodes.Moreover, the TuckER model primarily focuses on tensor decomposition representations between entities and relations, which restricts its ability to fully exploit complex relationships among entities. Therefore, we incorporated a nonlinear activation function, recognizing that the associations between entities and relations are often nonlinear. This inclusion allows the model to learn more complex and rich feature representations. Finally, we employed the improved TuckER model as a decoder for link prediction experiments.

To support our “Tea Suitability” research, we collected and proposed a tea knowledge graph dataset, TeaPle, encompassing numerous relationships. We conducted extensive comparative and ablation experiments on three datasets to address the challenge of selecting suitable populations for various tea types and to accurately evaluate the generalization of the improvements introduced by the GNN layer and nonlinear activation function. Experimental results demonstrate that our proposed GNN-FTuckER not only effectively addresses the “Tea Suitability Challenge” but also achieves outstanding performance on public datasets. Additionally, we evaluated the impact of different nonlinear activation functions on the link prediction model.

Despite the excellent achievements of GNN-FTuckER in the study of “tea variety and suitable population,” it still faces three challenges: 1) The TeaPle dataset needs to be expanded. While it covers six major tea types and 12 target populations, it does not encompass all global tea categories and representative populations. 2) The generalization ability of the GNN-FTuckER model needs improvement. For instance, on the FB15k-237 dataset, the H@1 metric is 27.4%, indicating room for improvement in capturing and understanding complex relationships. 3) As the graph data scale increases, the model’s computational complexity and resource consumption will rise significantly, directly impacting its applicability.

To address these challenges, future plans include continuously collecting tea data from different countries and regions to expand the dataset and increase the coverage of tea types and populations, which will address the first challenge. For the second and third challenges, we aim to optimize the model’s structure by adjusting hyperparameters, increasing model depth, or altering embedding dimensions, as well as exploring new graph neural network architectures or integrating advanced technologies to enhance feature extraction and representation capabilities. Additionally, we plan to extend the application of link prediction techniques to other agricultural domains, such as crop-soil suitability prediction, agricultural product quality assessment, disease prediction, and supply chain optimization. Beyond agriculture, link prediction algorithms can also be applied to other fields, such as predicting medical accidents.
